# Unbiased Model-Agnostic Metalearning Algorithm for Learning Target-Driven Visual Navigation Policy

**DOI:** 10.1155/2021/5620751

**Published:** 2021-12-08

**Authors:** Tianfang Xue, Haibin Yu

**Affiliations:** ^1^State Key Laboratory of Robotics, Shenyang Institute of Automation, Chinese Academy of Sciences, Shenyang 110016, China; ^2^Key Laboratory of Networked Control Systems, Chinese Academy of Sciences, Shenyang 110016, China; ^3^Institutes for Robotics and Intelligent Manufacturing, Chinese Academy of Sciences, Shenyang 110169, China; ^4^University of Chinese Academy of Sciences, Beijing 100049, China

## Abstract

As deep reinforcement learning methods have made great progress in the visual navigation field, metalearning-based algorithms are gaining more attention since they greatly improve the expansibility of moving agents. According to metatraining mechanism, typically an initial model is trained as a metalearner by existing navigation tasks and becomes well performed in new scenes through relatively few recursive trials. However, if a metalearner is overtrained on the former tasks, it may hardly achieve generalization on navigating in unfamiliar environments as the initial model turns out to be quite biased towards former ambient configuration. In order to train an impartial navigation model and enhance its generalization capability, we propose an Unbiased Model-Agnostic Metalearning (UMAML) algorithm towards target-driven visual navigation. Inspired by entropy-based methods, maximizing the uncertainty over output labels in classification tasks, we adopt inequality measures used in Economics as a concise metric to calculate the loss deviation across unfamiliar tasks. With succinctly minimizing the inequality of task losses, an unbiased navigation model without overperforming in particular scene types can be learnt based on Model-Agnostic Metalearning mechanism. The exploring agent complies with a more balanced update rule, able to gather navigation experience from training environments. Several experiments have been conducted, and results demonstrate that our approach outperforms other state-of-the-art metalearning navigation methods in generalization ability.

## 1. Introduction

Target-driven visual navigation has been a long-term goal in robotic community. It requires agent to navigate from an arbitrary location to a goal position [1], based on visual observations and user-specified targets [2]. Unlike traditional navigation paradigms such as SLAM [3] suffering from low data efficiency, mapless navigation paradigm tends to aggregate visual information into a meaningful state, in the hope of learning to solve the navigation problem implicitly through trials. With little prior knowledge of the tasks and implicit memorization of relationships between objects, an end-to-end mapless learning model is capable of mapping raw observations to values or actions and eliminating errors accrued from primary navigation engineering projects, including extracting observation features, building up map, ascertaining target location, and planning path [4]. Various deep reinforcement learning methods have been adopted into visual navigation field to construct such end-to-end learning architecture as DQN [5] and A3C [6]. After interacting with its surroundings, the navigation agent is capable of analyzing and inferring the aspects most relevant to the target to guide its navigation actions.

Recently, the main challenge existing in DRL-based navigation studies lies in generalization to unfamiliar environments. Usually this is known as the model imperfection issue. On account of the fact that DRL models are judged as black-box models with inalterable structure, they are susceptible to appearance changes and make quite poor performance in adapting to novel scenes [7]. Once a navigation model is fully updated based on a particular task, it cannot be employed to solve navigation problems of other targets or environments. To tackle this problem, plenty of works have been proposed such as scene-specific model [8], value and advantage saliency maps [9], learning spatial context [10], and multiview fusion technique [11]. However, none of these approaches can make the best of former experience and ensure good stability when configured for unfamiliar tasks.

Metalearning approach has been introduced as an effective way to improve the generalization capability of DRL model. According to prior metalearning studies in navigation field, an initial model can be trained across a variety of training tasks to acquire preliminary cognition of tasks and then further learns optimal parameters with few trials to achieve adaption in the new environment. Such adaption demands no further direct supervision but a few exploring iterations with novel environmental characteristics. A lot of metalearning-based approaches have shown promising results on improving generalization ability in visual navigation field. However, the main drawback of metalearning algorithms is that the primary model parameters are likely to be updated biased towards some particular tasks sampled in metatraining phase. The imbalance of certain classes in the dataset may also bring about bad influence on model performance [12]. In this case, the initial model can be prone to be overfitting to these specific scenes and may not effectively adapt to an unfamiliar environment with much deviation from these biased scenes. Hence, we try to avoid the initial model overtraining on some particular tasks, making sure that it can be more generalizable.

For this purpose, we propose an Unbiased Model-Agnostic Metalearning (UMAML) algorithm in this paper. Our approach is inspired by inequality measures defined in Economics, which was previously used to calculate regional differentiation characteristics of income or investment. Since each loss of training episode can be considered as an income for that task, we introduce this metric into visual navigation field to make the navigation model task-agnostic. According to our self-adapting learning architecture [4] derived from Model-Agnostic Metalearning (MAML) [13], we address the overfitting issue by means of metatraining an initial model which explicitly minimizes the inequality index of losses over tasks. This metalearner is required to update its parameters evenly, without overfitting to some particular tasks. The initial parameter can be rapidly regulated to the value that is most susceptible to variance of tasks. As novel tasks come up, these parameters keep on converging by few explorations in the unseen environment, until the model finally achieves adaption. Unlike entropy-based MAML approaches limited to discrete outputs from a model, our UMAML algorithm is quite suitable for the deep reinforcement learning mechanism, making it more amenable to end-to-end target-driven navigation tasks.

## 2. Related Work

### 2.1. DRL Models for Navigation

In recent years, deep reinforcement learning algorithm has been widely applied in visual navigation field. In contrast to conventional map-based approaches [14–16] or SLAM-based methods [3, 17, 18], deep reinforcement learning method plans navigation paths without a global cognition of running environment. Visual observation and spatial relation between agent and objects are all implicitly memorized into the network. Kim et al. [19] focused on extracting environmental features from visual observation, making integral navigation decisions. Zhu et al. [8] proposed a novel deep siamese actor-critic network to make navigation decisions directly depending on observed information and target image. Such siamese network with an A3C algorithm provides great compatibility for diverse targets. Gupta et al. [20] put forward a Cognitive Mapper and Planner for robot navigation, aiming to generate sequences of moving actions towards goals.

### 2.2. Vision and Language

As the visual features of target in the same category could be quite different, vocabulary and natural-language instructions have been gradually utilized to describe goals of navigation tasks. Misra et al. [21] aimed to combine raw visual observations and text-defined target as a joint feature. As the feature processed by LSTM and CNN, agent acquires a sequence of navigation instructions and moves in a 2D block scene. Wu et al. [22] focused on embodied agents which can complete a series of instructions in a simple maze world. Radwan et al. [23] proposed a visual navigation policy which is implemented on a wheeled-travelling robot. All the images observed by robot are all segmented in the light of semantic class so as to provide a better understanding of the contents in the surroundings, generating a more precise moving trajectory. However, all these studies fail to achieve generalization to previously unseen environments.

### 2.3. Metalearning

Nowadays, metalearning approaches have become much more popular for they optimize to learn experience from multiple training samples and accomplish new tasks quickly and efficiently. Common types of metalearning methods include (1) metric-based methods [24, 25], (2) memory-based methods [26, 27], and (3) gradient-based methods [28, 29]. In order to achieve rapid adaption to novel navigation tasks, many metalearning techniques have been adopted to construct self-learning architecture in visual navigation field. Anderson et al. [30] proposed a metalearning-based method to optimize navigation strategy by prerecorded prior exploration. With variability limited robot can adapt to new tasks after a few training episodes. Liu et al. [31] put forward a metacritic DRL method to learn parameterized skills, by which moving actions are instructed for unseen targets. Unlike these works, our approach relies on MAML algorithm to accomplish navigation tasks across untrained scenes, facilitating scene-domain generalization.

## 3. Proposed Method

Our goal is to train an unbiased navigation policy with the ability of rapidly achieving adaption in unfamiliar environments. In addition to adopting metalearning approach to construct a self-adaptive learning mechanism, our work provides new insights into inequality-minimization measures to balance the loss function values calculated in the metatraining phase, which avoids the primary model overperforming on a specific task. We will give a thorough description of our Unbiased Model-Agnostic Metalearning (UMAML) algorithm and discuss the characteristics of inequality measures in this section.

### 3.1. Problem Formulation

Since target-driven navigation model aims to acquire the shortest path from agent's current location to its target, the RL-based interactive process can be formulated as a tuple (*O*, *A*, *D*, *R*), regarded as partially observable Markov decision process [32]. Observations *O*={*O*_*T*_, *O*_*V*_}, combining target *O*_*T*_ in vocabulary form and visual observation of current state *O*_*V*_, are processed as the input of navigation model. Based on deep reinforcement learning, agent explores in the indoor scenes with sequence of actions *A*={*a*_1_, *a*_2_,…, *a*_*n*_}, where *a* includes 3 actions: moving forward, rotating left, and rotating right by 30 degrees.

In order to determine the shortest path from start to target location, the reward *R* : *O* ⟶ *R* is constructed as follows: reward 10 is received if agent arrives at the destination; reward -0.1 is obtained if a timestep has passed. Agent stops exploring until it navigates to its goal or a maximum number of actions have been taken. To evaluate generalization ability across scenes, we design a group of scenes *S*={*S*_1_, *S*_2_,…, *S*_*k*_} and target object class *G*={*G*_1_, *G*_2_,…, *G*_*m*_}. Each task is denoted by *τ* by such tuple *τ*=(*S*, *G*), with sets of scenes disjointed for the training tasks Γ train and the testing tasks Γ test. Agent keeps learning the action-value function *Q* and updates network parameters during training and testing procedure, until it adapts to the testing task.

### 3.2. Networks Architecture

**Figure 1:**
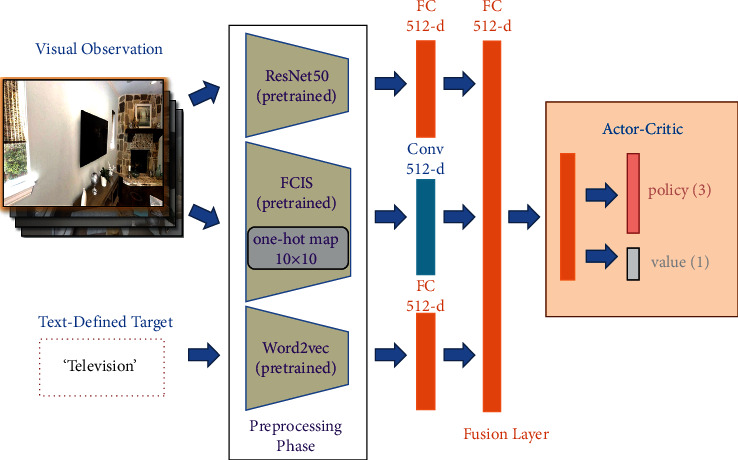
Our DRL-based network architecture. Compared to [6], we use FCIS and Word2vec model to extract semantic features and establish more efficient connection between goal and environment.

In Figure 1, the overview of the architecture is shown. Our DRL model is comprised of four modules: the ResNet50 module, the FCIS module, the Vocabulary-Encoding module, and the actor-critic module. The details of these networks are described as follows.

#### 3.2.1. ResNet50 Network

We select the ResNet50 [33] network adopted in [8] to extract features from the observed RGB frames. With the last FC layers removed, ResNet50 module is inserted to the front end of the model. All the parameters in the module are pretrained by ImageNet and then remain frozen during the whole learning phase, for the retraining procedure performed in some specific scenes may weaken the model's recognition performance and bring about more computational cost. After processing current observed frame and three previous frames, a combined visual feature is obtained and imported into a fully connected (FC) layer with ReLU activation, which finally outputs a 512-d feature as decision basis.

#### 3.2.2. FCIS Network

Fully Convolutional Instance-aware Semantic Segmentation network [34] is adopted to acquire a classifier, which predicts that each pixel's semantic class according to its likelihood score of the pixel belongs to specific object category, realizing instance-aware semantic segmentation. After filtering by nonmaximum suppression (NMS) with an intersection-over-union (IoU, 0.3 by default), the remaining Regions of Interest calculate their foreground masks by averaging likelihood scores of each map and weighting by classification scores, assigning one-hot semantic class id to each pixel [35]. Similar to ResNet50 module, the FCIS component is also pretrained and keeps its parameters unchanged during training and testing processes. When the agent comes to a novel state, current observation frame *O*_*V*_ will be passed into the FCIS module and output as a 10 × 10 semantic map *M*_*S*_, which indicates the semantic class of each observation region [7]. Finally, resized by four convolutional layers, a 512-d feature vector is received for navigation decision.

#### 3.2.3. Vocabulary-Encoding Network

In contrast to other visual navigation models such as that in [36], we utilize vocabulary to define the navigation target in favour of establishing semantic relation between observation and goal. Word2vec [37] model is configured as another input module translating a target into specific vectors with context relatedness encoded. Spacy toolkit is introduced to extract word-embedding, bringing about 300-d feature per target. As Figure 1 shows, word vectors are then combined with the output of ResNet50 and FCIS by a fusion layer which is similar to siamese neural network.

#### 3.2.4. Actor-Critic Network

With a 512-d joint representation from concatenated embedding of image and vocabulary vectors, the actor-critic module containing two fully connected layers generates the navigation decision that determines the action agent takes. The gradients in actor-critic and fusion module are back-propagated from the policy and value outputs back to the lower-level layers.

### 3.3. Unbiased Model-Agnostic Metalearning

In this study, a new task-agnostic approach has been proposed for learning balanced few-shot navigation policy. The main training mechanism is employed based on Model-Agnostic Metalearning (MAML) algorithm, which enables the primary model to solve new learning tasks using only a few training samples. However, the problem with the MAML approach is that, during the metatraining phase, the initial model is likely to favour some training tasks, particularly when metatesting tasks have much variance with those biased ones. As a solution, we introduce the inequality measures to prevent metalearner overperforming on training tasks.

#### 3.3.1. MAML-Based Approach for Visual Navigation

We develop a self-adaptive MAML-based algorithm to obtain proper initial parameter which can make rapid progress in navigating in new environments without overfitting. During the testing phase, any fine-tuned changes will bring about great modification on the task loss, resulting in acceleration of the convergence. According to MAML, we define that each task *τ* ∈ Γ train is sampled from metatraining dataset Dtr and metavalidation dataset Dval. The training objective of MAML is as follows:

(1) $$\begin{array}{c} \min\sum_{\tau \in \Gamma \text{ train}}\ell \left(\theta -\alpha \nabla_{\theta }\ell \left(\theta ,\text{Dtr}\right),\text{Dval}\right). \end{array}$$

The main goal of MAML work is to determine parameter *θ* that provides an optimized initial model for quick adjustment to novel tasks. To assure that the MAML mechanism can be applied into visual navigation field, we have made much modification in both metatraining and meta-adapting phases.

*(1) Metatraining Phase*. The initial model and its adaption process, presented by parametrized function *f*_*θ*_ with parameter *θ* and a loss function of *f*_*ϕ*_ with step-size hyperparameters *α*, *β*, *N*, are outlined in Algorithm 1. With sampling batches of tasks *τ*_*i*_ from training dataset, *K* trajectories *D*_*i*_ using *f*_*θ*_ in *τ*_*i*_ are collected as sequences of actions which indicate the current navigation policy. Such DRL procedure involves transition distribution *q*_*i*_(*X*_*t*+1_*|X*_*t*_, *a*_*t*_) with the loss function *ℓ*_*τ*_*i*__ taking the following form:

(2) $$\begin{array}{c} \ell_{\tau_{i}}\left(f_{\phi }\right)=-E_{x_{t},a_{t}∼f_{\phi },q\tau_{i}}\left[\sum_{t=1}R_{i}\left(x_{t},a_{t}\right)\right]. \end{array}$$

Then we employ the adapted parameters *θ*′ separately to collect new trajectories *D*_*i*_′. After all *τ*_*i*_ are processed, our initial adaptive model is updated as parameter *θ* shows.

*(2) Meta-Adapting Phase*. Algorithm 1 also presents that when the initial model is applied in unseen task, mini-batch of trajectories *D*^″^ is sampled. Once parameter *θ* is finally updated to *θ*^″^, our model can be able to navigate in the unfamiliar scenes. Generally the main idea is to integrate *K* rollouts from *f*_*θ*_, tasks *τ*_*i*_, and related rewards *R*_*i*_(*x*_*t*_, *a*_*t*_) as prior knowledge for fast generalization to testing tasks *τ*_*j*_.

#### 3.3.2. Inequality-Minimization for MAML

MAML and some other current metalearning approaches always have a certain flaw for achieving generalization that the metalearner may be biased towards particular training tasks. When dealing with the unfamiliar tasks in meta-adapting phase, the learning model may show unsatisfying performance for these tasks are dissimilar to the overtrained ones. Our work focuses on solving the problem of learning deviation and preventing metalearning model overfitting to a specific task, bringing about more effective update procedures across tasks.

In this study, we introduce Theil Index [38] into our self-adaptive approach to measure bias of tasks, which is considered as a financial statistic to measure the economic inequalities. Loss of each task *τ*_*i*_ can be regarded as the income for that task, and then, for our Unbiased Model-Agnostic Metalearning algorithm, the inequality of losses across training tasks should be minimized to balance their influences on the initial model. Hence, given unfamiliar tasks during meta-adapting phase, the model can be expected to be equipped with a better generalization ability by fine-tuning from an unbiased initial model with a few exploration trials in the new environments.

Here we first depict the paradigm of entropy from which Theil Index is derived. In the classification field, the initial model is preferred to calculate the entropy by sampling *x*_*i*_ over output probabilities *p*_*i*,*n*_ from *P*_*τ*_*i*__(*x*):

(3) $$\begin{array}{c} H_{\tau_{i}}\left(f_{\theta }\right)=-E_{x_{i}∼P_{\tau_{i}}\left(x\right)}\left[\sum_{n=1}^{N}p_{i,n}\ln\left(p_{i,n}\right)\right], \end{array}$$

where [*p*_*i*,1_,…, *p*_*i*,*N*_] is an output from softmax layer considered as the prediction of a classification task. This entropy can be not only maximized before the update of initial parameter but also minimized after the update to be utilized as a regularizer to search for the optimal parameter *θ*. However, when dealing with regression and reinforcement learning problems, there is no particular form of outputs to compute entropy. We need to introduce Theil Index as an alternative metric to ensure that the navigation model is task-agnostic based on loss or error functions.

Theil Index is derived from entropy in information theory, considered as the discrepancy between the maximum entropy of the data and an observed entropy. According to equation (3), Theil Index takes the following form:

(4) $$\begin{array}{c} T=\frac{1}{n}\sum_{i=1}^{n}\frac{y_{i}}{\bar{y}}\ln\left(\frac{y_{i}}{\bar{y}}\right), \end{array}$$

where *y*_*i*_ is the income of the *y*^th^ individual and $\bar{y}$ is the average income of all the individuals. *n* indicates the number of total individuals. In the visual navigation field, *y*_*i*_ and $\bar{y}$, respectively, represent the loss of *τ*_*i*_ and the average loss of all tasks *τ*. Hence, parameter *θ* is updated as follows:

(5) $$\begin{array}{c} \underset{\theta }{\min}\frac{E_{\tau_{i}∼P\left(\tau \right)}\left[\ell_{\tau_{i}}\left(f_{\theta_{i}}\right)\right]}{\lambda T}. \end{array}$$

Since achieving generalization across scenes includes agent generalizing to unknown scenes of same kind and different kind, we design Local Model and Global Model to be separately applied into these two scenarios.

For the Local Model, we define that Γ train is composed of tasks sampled from scene instances of one specific type, for example, bedroom01/bedroom02/bedroom03/bedroom04. As Algorithm 1 outlines, the trajectories are sampled, respectively, from different scenes of same kind and the initial model parameter is updated according to Theil Index in phase 11.

For the Global Model, we define that Γ train is composed of tasks sampled from scene instances of different types, for example, bedroom01/bathroom02/livingroom03/kitchen04. Since the bias of loss across different room instances and categories is required to be measured, we decompose Theil Index into *T*_*b*_ and *T*_*w*_:

(6) $$\begin{array}{c} T=T_{b}+T_{w}=\sum_{k=1}^{K}y_{k}\ln\left(\frac{y_{k}}{n_{k}/n}\right)+\sum_{k=1}^{K}y_{k}\left(\sum_{i\in g_{k}}\frac{y_{i}}{y_{k}}\ln\left(\frac{y_{i}/y_{k}}{1/n_{k}}\right)\right),\\ T_{b}=\sum_{k=1}^{K}y_{k}\ln\left(\frac{y_{k}}{n_{k}/n}\right),\\ T_{w}=\sum_{k=1}^{K}y_{k}\left(\sum_{i\in g_{k}}\frac{y_{i}}{y_{k}}\ln\left(\frac{y_{i}/y_{k}}{1/n_{k}}\right)\right), \end{array}$$

where *K* denotes the amount of scene category and *n* denotes the amount of scene instance. As *y*_*i*_ and *y*_*k*_ indicate the loss of the *i*_th_ instance and total loss of *k*_th_ scene category, *T*_*b*_ is calculated as inequality metrics to solve the problem of learning deviation across room instances, while *T*_*w*_ is adopted to measure bias of losses across room types. A batch of tasks *τ*_*i*_ are sampled from Γ_1_, Γ_2_,…, Γ_*K*_, which represent distinct kind of scene types.  Algorithm 2 shows that the initial model parameter is first tuned by *T*_*b*_ when agent explores in the different scene instances and then further updated by *T*_*w*_ when it is integrated with trajectories from other categories of scenes. The meta-adapting phase of Global Model is in accordance with that of Local Model as Algorithm 3 outlines, in which the optimal initial model gradually generalizes to the novel task.

Besides Theil Index, there are some other inequality measures that can be utilized to calculate regional differentiation characteristics of income or investment, such as Gini-Coefficient [39] and Variance of Logarithms [40]. Gini-Coefficient is defined as the half of the relative absolute mean difference, taking the following form:

(7) $$\begin{array}{c} G=1-\frac{1}{n}\left(2\sum_{i=1}^{n-1}W_{i}+1\right), \end{array}$$

where *W*_*i*_ indicates the percentage of the loss of the *i*_th_ instance. Compared to Theil Index, Gini-Coefficient is more susceptible to deviation around the middle of the distribution. Variance of Logarithms is defined as

(8) $$\begin{array}{c} V=\frac{1}{n}\sum_{i=1}^{n}\left[\ln\left(y_{i}\right)-\ln\;{\left(\prod_{i=1}^{n}y_{i}\right)}^{\left(1/n\right)}\right], \end{array}$$

where *y*_*i*_ indicates the loss of the *i*_th_ instance. Variance of Logarithms is more susceptible to deviation at the lower part of the distribution. Since Gini-Coefficient and Variance of Logarithms cannot be further decomposed, in the Global Model, these two metrics are utilized as they work in the Local Model. The comparison of navigation model performances using different inequality measures is analyzed in Section 4.

Our algorithm contributes to the generalization of deep reinforcement learning models by adopting inequality measures to estimate the task bias. As the state-of-the-art models generally ignore the great deviation between metatraining tasks and testing tasks, their metalearner has a great probability to excessively adapt to sampled tasks during training phase. Unlike conventional algorithms, our work introduces the deformation of such economic metrics to avoid the deviation of some specific tasks. By minimizing the inequality over the losses of sampled tasks in a batch (Local Model) and the losses of sampled tasks in the batch (Global Model), we increase the uncertainty of the initial model on different tasks to acquire an unbiased initial model, resulting in a better generalization ability compared to other metalearning navigation methods.

## 4. Experiments and Discussion

### 4.1. Experimental Setup

**Figure 2:**
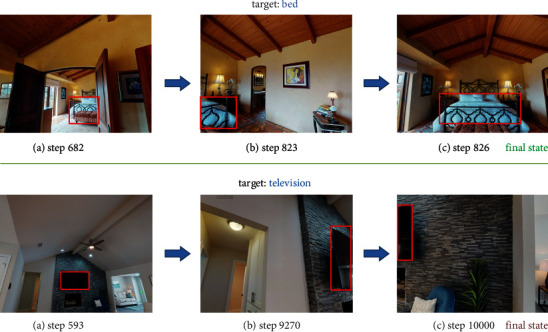
An example of exploring processes with two kinds of termination condition. The episode of navigating to bed ends when the agent observes the target clearly at the nearest viewpoint. Since in the other exploring process the agent fails to gain the full view of the television, the training episode is terminated at step 10,000.

We evaluate our model by testing on real-world 3D navigation dataset Matterport3D [41]. An exploring episode is determined to be finished once (1) the vocabulary-defined target appears within range of vision while agent reaches to the nearest viewpoint, since on many occasions the agent cannot directly get to the target, or (2) it has performed 10k moving steps failing to navigate to the destination. As Figure 2 shows, the exploring process of navigating to bed can be divided into several moving phases: (a) After plenty of moving steps taken, the observed image of step 682 first includes the full view of the target. As the agent has not arrived at the nearest viewpoint, it still needs to keep exploring in the scene. (b) With 823 moving actions performed, the agent gets to the nearest viewpoint, acquiring a partial view of the target. In this phase, the agent remains where it is and simply rotates its camera to catch the whole picture of the bed. (c) In step 826, the agent finally observes the ideal image and finishes the exploring episode. In contrast to the successful training episode, the exploring process of navigating to television fails to arrive at the nearest viewpoint; hence, the entire exploring process ends as 10k moving steps have been taken.

### 4.2. Evaluation Metrics

For comparison with other visual navigation models, we choose metrics presented by [23] to evaluate the model's navigation performance. The success rate (SR) is defined as

(9) $$\begin{array}{c} \text{SR}=\frac{1}{N}\sum_{t=1}^{n}S_{i}. \end{array}$$

Meanwhile, the Success weighted by Path Length (SPL) is calculated as follows:

(10) $$\begin{array}{c} \text{SPL}=\frac{1}{N}\sum_{t=1}^{n}\frac{S_{i}l_{i}}{\max\left(l_{i},e_{i}\right)}, \end{array}$$

where *N* is the number of running episodes. The shortest distance from the start viewpoint to the goal and the length of current episode are indicated by *l*_*i*_ and *e*_*i*_. *S*_*i*_ takes form as a binary vector declaring if agent succeeds in the *i*^th^ episode. In view of our model developing and reaching maturity after few explorations in the novel environment, we compute these two metrics after 100 episodes in the meta-adapting phase.

### 4.3. Generalization Performance

**Figure 3:**
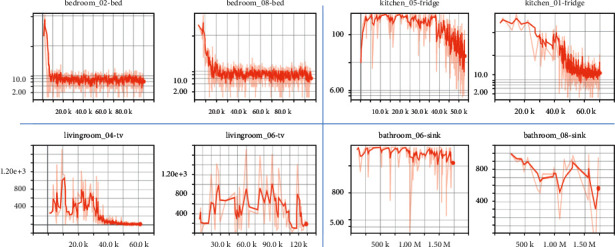
The steps-dependent learning curves of our MAML model without inequality minimization in metatesting phase. The *X*-axis indicates the number of moving steps taken; the *Y*-axis indicates the mean trajectory length of current episode as agent explores.

To demonstrate the significance of inequality minimization, especially the effect of Theil Index, we perform a series of navigation tasks using our MAML approach (Global Model) without drawing Theil Index into the model.  Figure 3 presents the learning curves in the meta-adapting phase with diverse targets and scenes. In the metatraining phase, the agent explores in 5 room instances of each scene type, while the navigation target remains the same. In the meta-adapting phase, the initial model is applied into 10 unfamiliar room instances to find the same target. The results shows that our MAML model without inequality minimization achieves primary convergence within average 20k exploring steps in unfamiliar bedrooms 02/08 and average 50k exploring steps in unfamiliar kitchen 01 and livingroom 04. However, in other unfamiliar room instances, our model fails to converge within 100 adapting episodes. Such unbalanced performance indicates that the initial metatrained model of conventional MAML algorithm could be overfitting to the spatial characteristics of bedroom scenes, such as room layout and illumination conditions, which leads to a great decline in navigation success rate of other scene types. In the following experiments, we will evaluate the generalization ability of our impartial model-agnostic metalearning algorithm in comparison with conventional MAML algorithm and other DRL navigation approaches.

Our proposed navigation model is trained based on Unbiased Model-Agnostic Metalearning algorithm as Section 3 describes. The learning procedure can be quite different between Local Model and Global Model.

#### 4.3.1. Local Model

During the metalearning phase, 4 navigation tasks of one specific scene type (bedroom) have been randomly selected to compose task set *τ*_1_ ~ *τ*_4_. For each task, 20 trajectories *D*_1_ ~ *D*_20_ are collected to calculate the loss function of same type *ℓ*_*τ*_1__ ~ *ℓ*_*τ*_4__ so as to metatrain parameter *θ* within *N* (500) batch of iteration. In the meta-adapting phase, the initial model is implemented on familiar target in unfamiliar scene of same type (e.g., bedroom), exploring until parameter *θ* finally converges to an optimal value.

#### 4.3.2. Global Model

During the metalearning phase, we choose 4 navigation tasks of four scene types (bedroom/kitchen/livingroom/bathroom) to compose task set *τ*_1_ ~ *τ*_4_. For each type of task, still 20 trajectories *D*_1_ ~ *D*_20_ are collected to calculate the loss function of each type *ℓ*_*τ*_1__ ~ *ℓ*_*τ*_4__. The adapted parameters *θ*′ are tuned according to their scene type and deployed, respectively, to obtain new trajectories *D*_1_ ~ *D*_4_. These trajectories from different scene types facilitate update of the primary model parameter. In the meta-adapting phase, the model is tested on random task from all four types of scenes.

**Figure 4:**
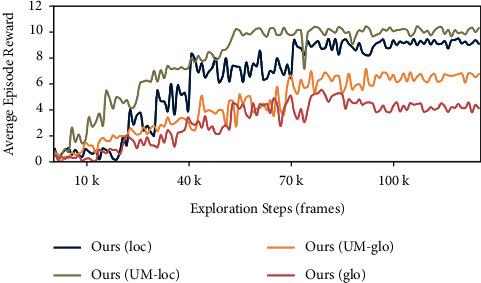
The learning curves of our initial MAML model Ours(loc)/Ours(glo) and UMAML model Ours(UM-loc)/Ours(UM-glo) applied into untrained bedroom scenes. The results demonstrate that Ours(UM-loc) and Ours(UM-glo) all achieve better performances than Ours(loc) and Ours(glo), as the UMAML initial models explicitly minimize the inequality of losses over sampled tasks.

Figure 4 shows the learning curves of our initial MAML models and UMAML models applied into untrained bedroom scenes. The result demonstrates that all of our models achieve preliminary convergence within average 70k actions taken. Due to its adaptability, our model's performance is quite superior to those of the models without metalearning mechanism, which needs to entirely retrain the model with average 500k–900k exploring steps to find the target. Additionally, compared to formal Local Model and Global Model without Theil Index adopted, the application of Unbiased Model-Agnostic Metalearning algorithm greatly improves average episode reward of successful episode by 50% increase. Since exploring in scenes of same type could bring about better navigation performance, our unbiased Local Model outperforms the unbiased Global Model by a narrow margin. Benefitting from inequality minimization, our UMAML models can be more effectively applied into novel environments.

Our model is further evaluated by comparison with other state-of-the-art navigation models. These models are partly reconfigured into our generalization experiments for comparison:

  TDVG: the primary model proposed by Zhu et al. [8] has similar architecture to ours but simply using RGB images to describe observation and targets.
  MPSL: this model achieves abstraction of targets by metacritic network so that agent can take advantage of parameterized skills to find unfamiliar goals [30].
  GCN: graph convolutional network is adopted in this model for incorporating the prior knowledge of semantic relation to analyze the most optimal trajectory [42].
  Our(loc): this model corresponds to the Local Model trained by MAML, performed in room scenes of one type.
  Our(glo): this model corresponds to the Global model trained by MAML, trained and tested in all four types of scene.
  Our(GC-loc): Our(GC-loc) is the Local Model trained by proposed Unbiased Model-Agnostic Metalearning algorithm, using Gini-Coefficient as the inequality measure.
  Our(VL-loc): Our(VL-loc) is the Local Model trained by proposed Unbiased Model-Agnostic Metalearning algorithm, using Variance of Logarithms as the inequality measure.
  Our(UM-loc): Our(UM-loc) is the Local Model trained by proposed Unbiased Model-Agnostic Metalearning algorithm, using Theil Index as the inequality measure.
  Our(GC-glo): Our(GC-loc) is the Global Model trained by proposed Unbiased Model-Agnostic Metalearning algorithm, using Gini-Coefficient as the inequality measure.
  Our(VL-glo): Our(VL-loc) is the Global Model trained by proposed Unbiased Model-Agnostic Metalearning algorithm, using Variance of Logarithms as the inequality measure.
  Our(UM-glo): Our(UM-glo) is the Global Model trained by proposed Unbiased Model-Agnostic Metalearning algorithm, using Theil Index as the inequality measure.

The cross-instance generalization performances of testing navigation models are shown in Table 1 with regard to SPL and SR. All training experiments are conducted on bedrooms in the trained dataset. We randomly select navigation tasks from the same training split with all the initial locations being at least 10 steps away from the targets. In the testing phase ,the trained models are required to navigate in four unfamiliar scene instances of bedroom (bedroom01/bedroom02/bedroom03/bedroom04). It can be seen that Our(loc) and Our(UM-loc) have outperformed the baselines with large margins. The success rate of our model was 30% to 40%, nearly 25% higher than others.  Table 2 shows the cross-scene generalization performances of testing models as all the training and testing tasks are performed in all four different scene types (bedroom/kitchen/livingroom/bathroom). The success rates of Our(loc) and Our(UM-glo) increase by about 15% compared to GCN. Such discrepancy is likely to be caused by the limitation of structural framework and training approach. Unlike UMAML, TDVG can be considered as a nonadaptive model that could always generate invalid navigation decisions under inexperienced situation. MPSL and GCN are quite unstable, since their task features abstracted lose availability as the appearance of scene changes.

Most notably, Our(UM-loc) and Our(UM-glo) greatly decrease the standard deviation of SPL/SR by 46%/58% and 23%/56%, respectively. These results demonstrate that our inequality minimization mechanism successfully reduces the bias of loss across different scene instances and categories, which proves that the introduction of Theil Index solves the problem of learning deviation and prevents metalearning model overperforming on some specific tasks. Unlike other models' success rate varying considerably in different scenes, our UMAML approach maintains relatively balanced performances across distinct tasks, guaranteeing the navigation stability to a certain extent. It is worth mentioning that Our(UM-loc) achieves better result in standard deviation than Our(UM-glo). The results indicate that, compared to the Local Model, even minimizing the inequality between task losses, there is still a chance that variance across scene types leads to a slight bias towards particular tasks.

Gini-Coefficient and Variance of Logarithms have also made a great contribution to improving the generalization ability of MAML model. Considering the agent navigating in the same scene type, Our(GC-loc) and Our(VL-loc) achieve similar SPL, SR, and standard deviation to Our(UM-loc), which verifies that these two metrics can substitute for Theil Index to measure the inequality index of losses over tasks in the Local Model. However, when dealing with the deviation in both scene instances and scene types, Theil Index outperforms other inequality measures due to its decomposability. As there are also some other inequality measures such as Generalized Entropy Index [43] and Atkinson Index [44] that are capable of solving bias problems in the DRL navigation field, we will conduct more experiments to validate their availability.

**Figure 5:**
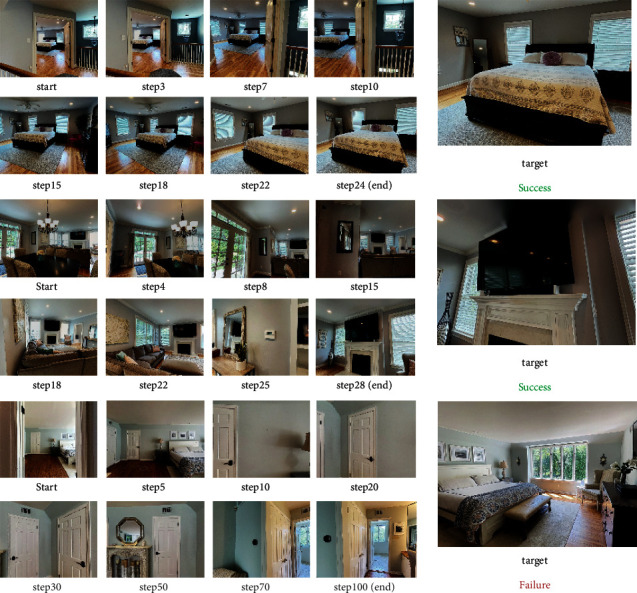
Visualization of the three trajectories to reach targets from start images. The first two navigation tasks have been successfully accomplished, while the third exploring process ends in failure due to task-irrelevant interference factors such as doors and corridors.

In addition, we observe that our model's navigation performance takes on a descending trend during the adapting process as occlusion factors gradually appear in the observation such as doors, mirrors, and corridors. In this case, there is a high probability that the agent gets stuck or wanders around without making progress. See Figure 5 for three front-view trajectories generated by our Our(UM-glo) method. For the first two navigation tasks in unfamiliar bedroom and livingroom, the agent arrives at the target location within 30 steps. However, in the third scenario, the navigation tasks of bed fail to be accomplished within 100 steps as the view has been blocked by door frames and walls. Considering navigation efficiency, all the real-world scenes sampled for experiment should be split into spacious areas to get rid of interference factors.

## 5. Conclusion

In this paper, we present an Unbiased Model-Agnostic Metalearning (UMAML) algorithm for learning target-driven navigation policy. Compared to most state-of-the-art visual navigation approaches, we introduce Theil Index, an inequality measure used in Economics, as an alternative metric to measure the bias across tasks. The key idea is to train the metalearner by means of explicitly minimizing the inequality index of losses over tasks, so that the metalearner can update its parameters evenly, avoiding overfitting to some particular tasks. To evaluate its performance, several experiments have been conducted on finding familiar targets in unfamiliar scenes. As results illustrated, our model consistently outperforms existing visual navigation approaches and maintains satisfying performance no matter how the instance or category of scene changes. In the future, we will pay more attention to other vital observation features such as depth to learn navigation experience in a more efficient way and reconfiguration of current metalearning mechanism to achieve better generalization.

## Figures and Tables

**Algorithm 1:**
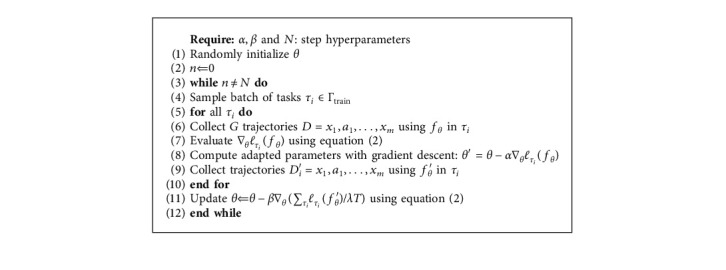
Local Model: metatraining phase.

**Algorithm 2:**
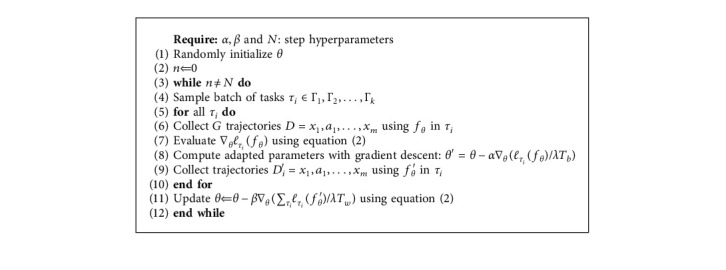
Global Model: metatraining phase.

**Algorithm 3:**
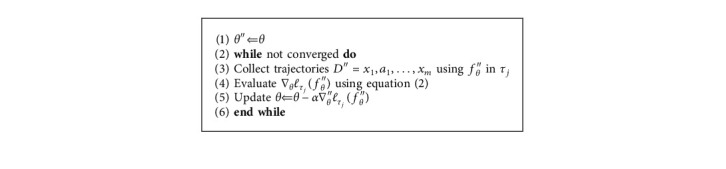
Local/Global Model: meta-adapting phase.

**Table 1:** Comparison results of standard deviation for SPL and SR in the same scene type.

Scenario	Method	Scene instance								SD	
		bedroom01		bedroom02		bedroom03		bedroom04			
		SPL	SR	SPL	SR	SPL	SR	SPL	SR	SPL	SR
Trained target in unseen environment	TDVG	8.56	13.7	0.81	4.11	0.56	3.89	9.41	15.7	4.81	6.23
	MPSL	6.87	10.8	11.4	19.9	2.51	8.44	3.41	11.2	4.03	5.02
	GCN	7.19	12.1	16.4	25.2	15.9	22.9	17.2	31.6	4.68	8.11
	Ours(loc)	13.4	**42.3**	**19.8**	**38.6**	19.4	**41.1**	11.7	29.4	4.13	5.84
	Ours(GC-loc)	11.5	33.6	17.7	30.2	15.1	29.7	18.9	38.6	3.27	4.10
	Ours(VL-loc)	**15.1**	40.1	15.0	36.2	14.9	36.5	**19.8**	**40.2**	2.40	**2.19**
	Ours(UM-loc)	14.4	40.2	17.9	38.4	**19.6**	36.8	18.3	34.5	**2.22**	2.42

**Table 2:** Comparison results of standard deviation for SPL and SR in the different scene types.

Scenario	Method	Scene instance								SD	
		bedroom		kitchen		livingroom		bathroom			
		SPL	SR	SPL	SR	SPL	SR	SPL	SR	SPL	SR
Trained target in unseen environment	TDVG	1.42	9.14	9.81	17.7	15.6	**30.8**	5.45	13.9	6.07	9.29
	MPSL	4.26	12.3	10.6	22.8	2.47	9.34	10.5	25.8	4.21	7.97
	GCN	**11.4**	25.8	15.1	32.5	5.43	12.1	14.3	28.9	4.38	8.91
	Our(glo)	9.25	20.5	**17.4**	**36.8**	8.56	18.4	12.1	31.3	4.01	8.76
	Our(GC-glo)	8.34	15.4	11.4	28.6	12.1	20.4	**17.7**	**36.6**	3.90	9.32
	Our(VL-glo)	7.2	24.7	14.5	31.4	9.81	19.1	14.8	27.2	3.70	5.14
	Our(UM-glo)	10.1	**33.6**	15.5	35.7	**13.5**	26.8	17.2	34.7	**3.05**	**3.85**

## Data Availability

The data used to support the findings of the study are available from the corresponding author upon request.
